# Influence of Montmorillonite Organoclay Fillers on Hygrothermal Response of Pultruded E-Glass/Vinylester Composites

**DOI:** 10.3390/polym16152157

**Published:** 2024-07-29

**Authors:** Vistasp M. Karbhari

**Affiliations:** 1 Department of Civil Engineering, University of Texas, Arlington, TX 76006, USA; vkarbhari@uta.edu; 2 Department of Mechanical and Aerospace Engineering, University of Texas, Arlington, TX 76006, USA

**Keywords:** pultrusion, filler, calcium carbonate, montmorillonite, organoclay, moisture, durability, hygrothermal, immersion, tension, flexure, short beam shear

## Abstract

Pultruded fiber reinforced polymer composites used in civil, power, and offshore/marine applications use fillers as resin extenders and for process efficiency. Although the primary use of fillers is in the form of an extender and processing aid, the appropriate selection of filler can result in enhancing mechanical performance characteristics, durability, and multifunctionality. This is of special interest in structural and high voltage applications where the previous use of specific fillers has been at levels that are too low to provide these enhancements. This study investigates the use of montmorillonite organoclay fillers of three different particle sizes as substitutes for conventional CaCO_3_ fillers with the intent of enhancing mechanical performance and hygrothermal durability. The study investigates moisture uptake and kinetics and reveals that uptake is well described by a two-stage process that incorporates both a diffusion dominated initial phase and a second slower phase representing relaxation and deterioration. The incorporation of the organoclay particles substantially decreases uptake levels in comparison to the use of CaCO_3_ fillers while also enhancing stage I, diffusion, dominated stability, with the use of the 1.5 mm organoclay fillers showing as much as a 41.5% reduction in peak uptake as compared to the CaCO_3_ fillers at the same 20% loading level (by weight of resin). The mechanical performance was characterized using tension, flexure, and short beam shear tests. The organoclay fillers showed a significant improvement in each, albeit with differences due to particle size. Overall, the best performance after exposure to four different temperatures of immersion in deionized water was shown by the 4.8 mm organoclay filler-based E-glass/vinylester composite system, which was the only one to have less than a 50% deterioration over all characteristics after immersion for a year in deionized water at the highest temperature investigated (70 °C). The fillers not only enhance resistance to uptake but also increase tortuosity in the path, thereby decreasing the overall effect of uptake. The observations demonstrate that the use of the exfoliated organoclay particles with intercalation, which have been previously used in very low amounts, and which are known to be beneficial in relation to enhanced thermal stability, flame retardancy, and decreased flammability, provide enhanced mechanical characteristics, decreased moisture uptake, and increased hygrothermal durability when used at particle loading levels comparable to those of conventional fillers, suggesting that these novel systems could be considered for critical structural applications.

## 1. Introduction

Once used almost exclusively in the aerospace, marine, and automotive areas, fiber reinforced polymer (FRP) composites are increasingly being used in civil infrastructure, electrical power, and offshore/marine applications. In many applications, FRP composites present effective alternatives to conventional materials while enhancing performance characteristics through material–configuration–process tailoring and designed multifunctionality. Of all the processes used to fabricate FRP composites, pultrusion is the only one capable of processing continuous lengths of constant cross-section/profile with a very high degree of uniformity in performance characteristics while also controlling fiber architecture, enabling the addition of specific fillers and additives to the overall material system and high rates of production. Extensive reviews of applications of the pultrusion process and of pultruded composites in structural applications can be found in [1,2,3] and hence will not be repeated herein. Beyond their use in civil infrastructure systems as reinforcement in concrete replacing steel rebar and cables, and as replacements for metal and timber profiles, pultruded components are used extensively in offshore platforms, floating facilities, marine and offshore terminals, and subsea facilities in applications ranging from beams, grids, and decks, to umbilical and flexible pipes for deep water installations and risers due to their lightweight, resistance to corrosion, and insulative properties, as well as potentially lower life cycle costs. Pultruded profiles have also found extensive use in the power and transmission industry in applications ranging from cross-arms, pylons, and poles to insulators [4,5] and transformer housing units in addition to performance and reliability critical structural components such as access ways, support beams, and high elevation catwalks, in all of which the resistance to corrosion, heat damage, thermal warping, and electrical arcing provide significant advantages over conventional materials. In addition, pultruded sections and profiles are increasingly being used in wind turbine applications for components such as blades, spars, and caps [6,7,8,9].

In addition to fibers, fillers such as calcium carbonate, calcium sulfate, alumina trihydrate, talc, kaolin, and carbon-black, amongst others, are added to the resin to lower overall product costs by taking the place of the more costly resin and serve as resin extenders [10]. Fillers are also used to enhance process efficiency including preventing the adhesion of the resin to the die, lowering the exothermic peak, controlling the cure profile, reducing microcracking in the composite, and increasing line speeds and productivity [11,12]. In addition, fillers can enhance surface finish as well as suppress flame and smoke generation, and reduce porosity [13,14], with smaller size particles having better bonds with the resin and greater distribution through the composite [14]. While fillers such as calcium carbonate and talc have been used at particle loading levels as high as 50% of the total resin by weight [10], care must be taken to ensure that its addition does not result in decreases in mechanical performance and durability characteristics, especially as related to exposure to humidity and solution. Because of differences in particle size, surface characteristics, and chemistry, the results in a composite are seen to vary widely with type of filler. Manjunath et al. [15] showed improvements in mechanical properties at the 10% weight loading of alumina silicate and alumina trihydrate filled glass fiber composites, whereas He et al. reported increases in compressive strength using nano-CaCO_3_ [16] and Gupta et al. [17] reported a decrease in tensile strength with an increase in calcium carbonate loading. Kiran et al. [18], in comparison, reported increases in properties for a range of fillers at 10% weight loading with decreases at higher levels of loading. Boukhili et al. [12], however, reported that increasing filler content from 20% to 40% resulted in a very small decrease in shear strength, with the flexural characteristics being insensitive to loading level. Despite the extensive use of fillers in fiber reinforced composites, especially in pultrusion, there is a scarcity of data on the effects of the range of filler type, size, and loading on resulting characteristics including moisture uptake [11], and of the corresponding overall hygrothermal response over extended periods of time.

The increasing use of composites in structural applications with high performance requirements including enhanced resistance to flammability, increased thermal stability, and decreased solvent uptake has resulted in the consideration of organoclays such as montmorillonite as fillers [19,20]. Montmorillonite is a natural clay mineral with a dioctahedral 2:1 phyllosilicate with 2 O-Si-O tetrahedral sheets sandwiching an O-Al(Mg)-O octahedral sheet with interlayer space [19]. The layers are held together by van der Waals forces to form particles which in turn aggregate to form secondary nanometer and micrometer scale particles [21,22,23]. These silicate particles can provide enhanced thermal stability, flame retardancy, and decrease flammability and moisture uptake [23] when added to resins, but in general sacrifice strength, elongation, and toughness [24] unless they are appropriately exfoliated, whereby significant improvements in tensile strength and a modulus of up to 74% and 67%, respectively, from a 2.5 to 7.5 weight percent addition [25], and compressive strength of 22–36% at 3 to 5% loading [26] and shear strength [14,27] have been reported, over that of the neat resin. When added to fiber reinforced composites, a decrease in glass transition is noted [20,28,29], accompanied by an increase in water uptake [20,28]. At the low levels of loading used, there are conflicting results of increases [28] and no change [29] in the flexural characteristics of fiber reinforced composites with organoclay filler particles.

The levels of particle loading in these composites, however, are too low to be effective in commercial pultruded composites wherein loadings in the 15–25% range are normal [30]. Despite the significant potential in structural applications, as well as in high voltage applications, there is a lack of data related to the use of organoclay particles at higher loading levels comparable to that of calcium carbonate, which has traditionally been used in infrastructure and pylon type applications. In addition, data related to moisture uptake in this type of system are lacking, which is similar to the situation in other particle filled fiber reinforced composites [31]. These data are essential for systems designed for lifetimes exceeding 50 years [32]. It is thus critical to obtain a comprehensive understanding of the moisture uptake and diffusion characteristics of these particle filled fiber reinforced composites [31] and the resulting effects on mechanical properties [20].

This paper reports on the experimental findings of an investigation related to the effect of three particle sizes and two loading levels of montmorillonite organoclay fillers on mechanical performance as determined through tensile, flexural, and short beam shear tests on E-glass/vinylester pultruded composites, including after hygrothermal exposure for a period of one year. To provide a comparison with the more conventional system specimens with calcium carbonate filler at the same loading level, 20%, are also included as a baseline.

## 2. Materials and Test Methods

An epoxy vinylester (Derakane Momentum 640-900), based on a bisphenol-A epoxy with 30% styrene specifically designed for pultrusion, was used. The resin has a viscosity of 900 cps with a gel time of 2.5 min at 190 °C, which represents the high temperature in the three-stage pultrusion process. The neat resin had an average tensile strength and modulus of 90 MPa and 3.5 GPa, respectively, and an average flexural strength and modulus of 160 MPa and 4 GPa, respectively. The fiber reinforcement consisted of Hybon 2001 E-glass fiber roving of 23 m/kN with a surface veil and continuous strand mat layer on the two surfaces. The pultrusion line was run at 91 cm/min with a resulting fiber volume fraction of 40% ± 2.5%, determined through burnoff procedures. Montmorillonite organoclay particles of 0.6 μm, 1.5 μm, and 4.8 μm, were added to the resin, resulting in 20% by weight of resin loading levels and the resulting system was then used as normal in the pultrusion process. An additional system with a 10% level was also used with the 1.5 μm particles. A set of specimens with 20 wt% of calcium carbonate as filler was also prepared to provide a basis for comparison since calcium carbonate is widely used as a filler in E-glass pultruded products. The level of filler content was selected to correspond to the standard levels used in pultrusion of 15–20% by weight of the neat resin [30]. For ease of reference, through this paper, the systems are designated as *OC: particle size/loading level* for the E-glass/vinylester with the organoclay fillers, and CaCO_3_ for the E-glass/vinylester composite with calcium carbonate fillers. Pultruded samples were of 100 mm nominal width and 4 mm nominal thickness. Prior to testing, all specimens were preconditioned at 23 °C and 30% relative humidity for 60 days to provide a uniform baseline.

Moisture uptake was studied using specimens with a 25.4 mm × 24.5 mm size cut from the pultruded strips and immersed in deionized water baths at controlled temperatures of 23 ± 1 °C, 40 ± 1 °C, 55 ± 1 °C, and 70 ± 1 °C for a period of 12 months. These temperatures were used to enable the assessment of response to both temperature and to enable the later use of time–temperature superposition principles for the prediction of long-term response. Specimens were placed in the baths to ensure no contact or overlap between adjacent specimens so that the exposure was consistent and over the full specimen. Test specimens were removed from the baths at periodic intervals with padded tweezers (to ensure that the pressure on the surfaces was minimized and that no dirt/oils/substances from human hands could inadvertently contaminate the specimens and baths), patted dry with tissue paper, and then weighed to measure changes due to the period and temperature of immersion, after which they were reinserted in the water baths. The procedure, including time for the operation, was standardized to ensure uniformity and consistency in the experiments. A minimum of 5 specimens were used for each measurement.

Mechanical characterization was conducted through tension, short beam shear (SBS), and flexural tests with five specimens used for each time period of exposure condition. Tensile tests were conducted on specimens of 255 mm × 25.4 mm with a gauge length of 180 mm following ASTM D3039 [33] at a crosshead speed of 2 mm/min. Flexural tests were conducted following ASTM D790 [34] in four-point bend mode in the 1/3 rd span configuration on specimens cut in the longitudinal direction (i.e., fiber reinforcement being along the test span), with a width of 12 mm and a 16:1 span to thickness ratio. The flexural strength is determined as follows:

(1) $$\sigma_{f}=\frac{PL}{bd^{2}}$$

where *P* is the failure load, and *L*, *b*, and *d*, are the support span, width, and thickness of the specimen, respectively. Short beam shear tests were conducted in both longitudinal and transverse directions following ASTM D2344 [35], with length and width being six and two times the thickness, respectively. The short beam shear strength is determined as follows:

(2) $$\sigma_{SBS}=0.75\frac{P}{bh}$$

where *h* is the specimen thickness. A minimum of five specimens were tested in each of the characterization modes for each test period. The intent was to not only characterize the as-received material as a function of particle type, size, and loading level, but also after periods of hygrothermal exposure to assess overall durability. It was expected that while the effects of fillers would be seen through the tensile tests, the moisture uptake effects would be better assessed through interfacial effects, which were likely to be greater and better seen through the flexural and short beam shear tests.

## 3. Results and Discussion

### 3.1. Moisture Kinetics

**Figure 1:**
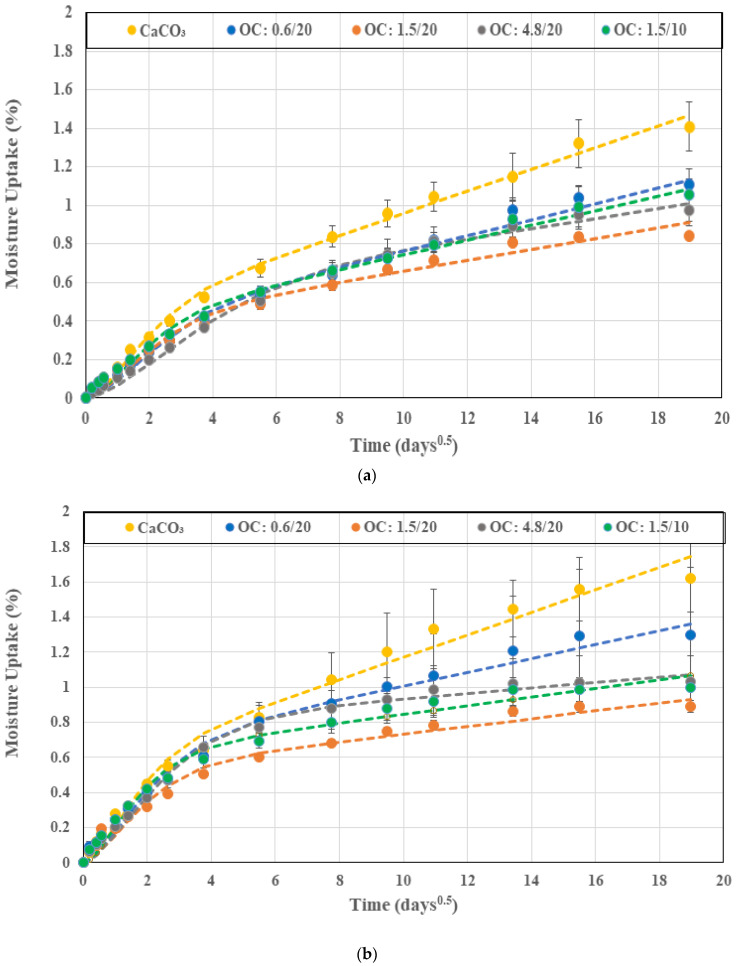

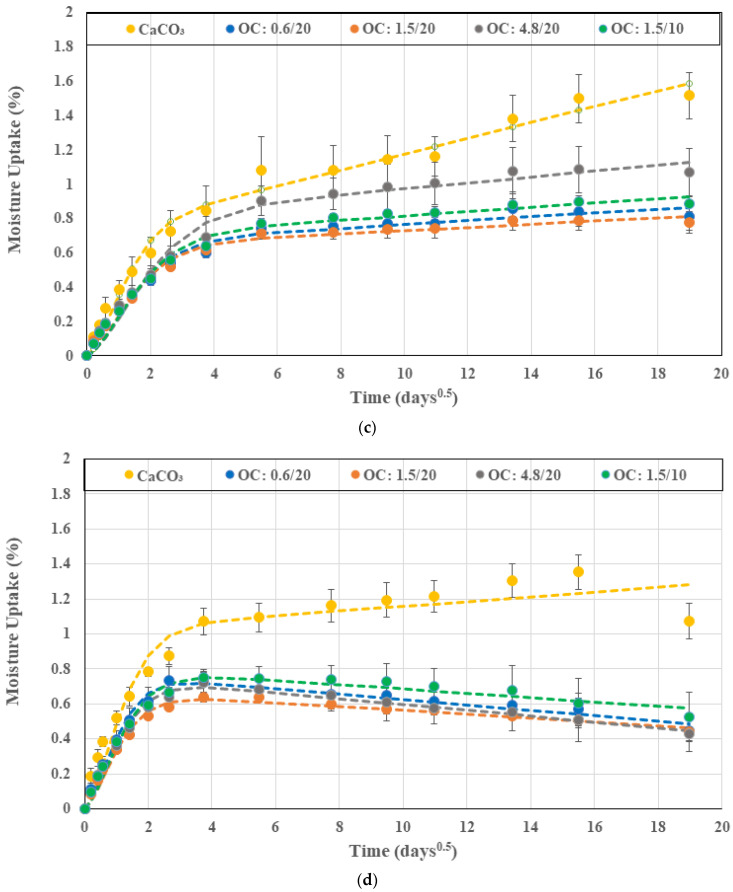
(**a**) Moisture uptake in deionized water at 23 °C. (**b**) Moisture uptake in deionized water at 40 °C. (**c**) Moisture uptake in deionized water at 55 °C. (**d**) Moisture uptake in deionized water at 70 °C. Dashed lines show predictions of the two-stage model.

**Figure 2:**
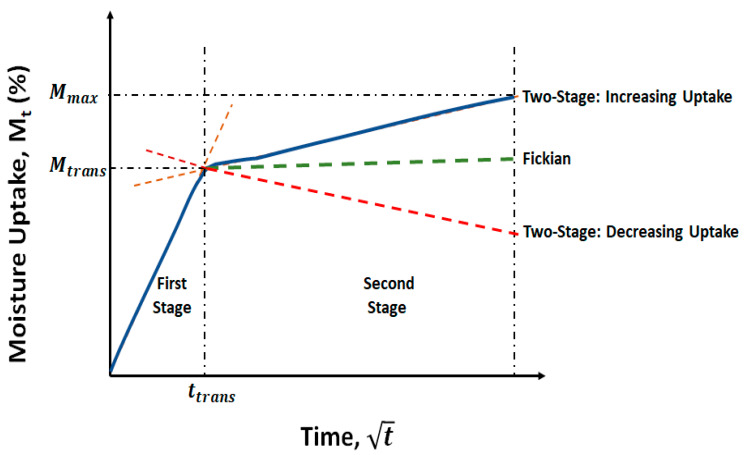
Schematic of moisture uptake models.

The relative moisture change (*M*%) for specimens immersed in water was determined as follows:

(3) $$M\left(\%\right)=\frac{M_{t}-M_{0}}{M_{0}}\times 100$$

where *M_t_* is the mass measured after time *t* of immersion and *M*_0_ is the initial mass measured just prior to immersion of samples in the baths of deionized water. Moisture uptake traces for the five different systems (four with organoclay fillers and one with the calcium carbonate filler) as a function of the square root of time are shown in Figure 1a–d at temperatures of immersion of 23 °C, 40 °C, 55 °C, and 70 °C, respectively. As can be seen, the uptake increases with time for all specimens at the first three temperature levels. At the level of 55 °C, a minor drop is noted over the final period of measurement, whereas at the highest temperature of immersion, 70 °C, the mass is seen to initially increase and then decrease. The decline occurs earlier for specimens with the organoclay fillers, while the specimen with the calcium carbonate fillers continues to gain mass until past 240 days, after which there is an abrupt drop. It is emphasized that in the first set of responses, saturation is not attained over the full period of immersion, with mass continuing to increase due to sorption. In the second set, the decrease after the attainment of a peak suggests mass loss due to the decomposition of low-molecular weight constituents, leading to the leaching of species into the solution, as suggested earlier [36,37], as well as the potential loss of the filler itself after interfacial deterioration at the higher temperatures. Both these responses do not follow the simplistic Fickian process that is most often used to describe moisture uptake in composites. Small molecule diffusion in polymers and composites as well as moisture-induced changes in the resin and interface levels, including from deterioration and relaxation processes, which are slower than the standard diffusion process, often cause uptake to continue increasing without saturation levels being attained over extended periods of exposure [38,39] or resulting in decreases after the attainment of a peak. The combination of the concentration gradient-driven Fickian diffusion and other slower time-dependent processes is thus better characterized by a two-stage model in which the differential rate for slow relaxation and moisture-uptake-induced deterioration processes can be described in line with Bagley and Long [40], following the initial work of Bao et al. [38], and as applied to wet layup composites [41] as follows:

(4) $$\frac{M_{t}}{M_{trans}}=\left(1+k\sqrt{t}\right)\left\{1-exp\left[-7.3{\left(\frac{Dt}{h^{2}}\right)}^{0.75}\right]\right\}$$

where *M_trans_* is the moisture uptake level representative of the transition between the two stages as shown in Figure 2, *k* and *D* are the relaxation/deterioration and diffusion coefficients, respectively, and *h* is the specimen thickness. It is noted that when *k* is equal to 0, Equation (4) reverts to the Fickian form of diffusion modeled by Shen and Springer [42]:

(5) $$\frac{M_{t}}{M_{\infty }}=1-exp\left[-7.3{\left(\frac{Dt}{h^{2}}\right)}^{0.75}\right]$$

where *M_∞_* is the saturation uptake level. A positive value of *k* in Equation (4) represents a relaxation dominated mode wherein moisture uptake continues to increase with time, whereas a negative value represents deterioration wherein the overall mass level decreases after the peak, indicating a competition between mass gained due to uptake and lost due to the leaching of low molecular weight species and interfacial and filler material. The two-stage model described in Equation (4) is seen to fit the experimental data extremely well, as shown by the dashed lines in Figure 1. The only exception to this is the case of the system with calcium carbonate filler immersed at 70 °C, where there is a sudden drop in mass after 240 days of immersion. This suggests a fairly rapid loss due to interfacial cracking and material loss, which does not follow the predominant mechanisms seen in all other specimens. The increase in uptake with time has been noted earlier in pultruded E-glass fiber/epoxy specimens with montmorillonite fillers [20] and in pultruded E-glass/isophthalic polyester composites [43,44], amongst others.

Moisture uptake characteristics and kinetics can be described through an assessment of four key parameters: (1) the maximum uptake level, *M_max_*, acknowledging that saturation was not attained for any of the specimens and that in some cases the competition between mass loss due to leaching and deterioration can offset some level of uptake; (2) the first stage diffusion coefficient, *D*; (3) the second stage relaxation/deterioration coefficient *k*; and (4) the level of moisture uptake at the nominal transition between the two stages, *M_trans_*. Since the levels of *M_max_* vary with the system and temperature of immersion, it is perhaps more illustrative to assess the transition ratio, *M_trans_/M_max_*, since this indicates the extent of each phase and can yield greater comparative information than just from the transition point [45]. It is of interest to compare responses based on three sets of data: (a) the particle size of the organoclay filler, (b) the level of loading of the organoclay for the 1.5 μm particles, and (c) the calcium carbonate and organoclay fillers at 20 wt% of resin mass loading level.

**Figure 3:**
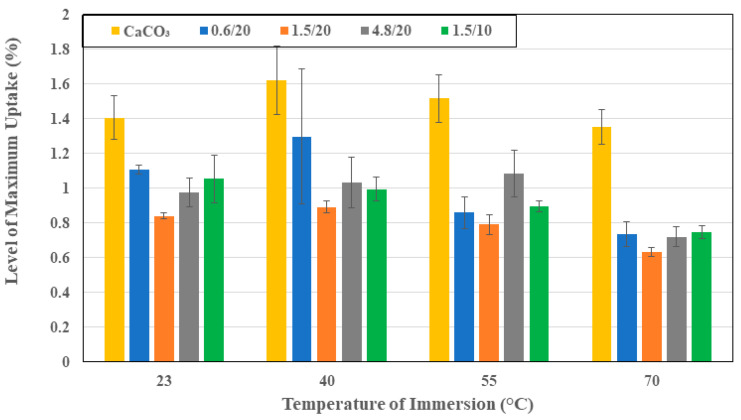
Maximum uptake as a function of filler and temperature of immersion.

As seen in Figure 1a–d, specimens with organoclay fillers all showed significantly lower uptake levels than the specimen with calcium carbonate fillers, emphasizing the role of the organoclays in creating a torturous path for moisture molecules through better interfacial bonding and significantly increased interfacial surface area due to the layered nature of the organoclays. The decrease in uptake levels had been previously reported by Becker et al. [46]. Figure 3 shows the overall comparison of maximum uptake levels as a function of the temperature of immersion for the different specimen types, with the uptake level being the lowest for 1.5 μm organoclay at 20% loading, with uptake levels being 40.2%, 45%, 47.8%, and 53.1% less than those with the calcium carbonate filler at the four different temperatures of 23 °C, 40 °C, 55 °C, and 70 °C, and with the gap increasing with temperature. This highlights the stability due to the use of organoclays as well. Uptake levels decrease with an increase in particle size from 0.6 μm to 1.5 μm, and then increase at 4.8 μm, emphasizing the existence of an optimal size level at which the exfoliated clay structures are most efficient as barriers against water transport. An increase in particle size beyond this perhaps results in a level of non-uniform dispersion or of exfoliation being hindered, as hypothesized earlier [32,47,48,49]. It is interesting to note that for all materials, the increase in the temperature of immersion from 23 °C to 40 °C results in an increase in uptake with the rate of increase being the highest for the composite with CaCO_3_ fillers and decreasing with particle size of the organoclay fillers. The increased level of scatter in the maximum uptake levels at 40 °C for the system with CaCO_3_ fillers and the system with 0.6 μm organoclay fillers is due to local microcracking and consequent wicking in some specimens, which result in larger variation. A further increase in the temperature of immersion to 55 °C results in increases until 240 days with minor decreases in the uptake level thereafter, suggesting a combination of both curing and polymer morphology changes that result in a lower uptake and a level of mass loss due to leaching. This is also the reason for the uptake at 40 °C being higher than at 55 °C since the competition between phenomena and mass loss results in a lower level of apparent uptake. It should be noted that the decrease in uptake level for the composites with organoclay fillers decreased with the increase in particle size with drops of 3.23%, 1.85% and 1.52%, for the systems with 0.6 μm, 1.5 μm, and 4.8 μm particles, respectively. In all cases, the decrease in uptake is within the scatter bounds. At the highest temperature of 70 °C, all systems showed the attainment of a peak with larger drops thereafter, with the level of uptake of the composites with organoclay particles being fairly similar with the lowest level being for the 1.5 μm particles at 20% loading. The system with calcium carbonate fillers showed a dramatic drop after 240 days of immersion. The decreasing trend at 70 °C is due to the leaching of low molecular weight species as well as that of fillers in areas of local microcrack-induced deterioration [36,37]. The comparison of the composites with the 1.5 μm particles at the two filler loading levels (10% and 20%) showed better performance, i.e., lower uptake levels at the higher loading level of 20%, with the composites at the 10% loading level having 25.2%, 11.6%, 13.4%, and 17.9% greater uptake at the four temperature levels of 23 °C, 40 °C, 55 °C, and 70 °C, respectively.

**Figure 4:**
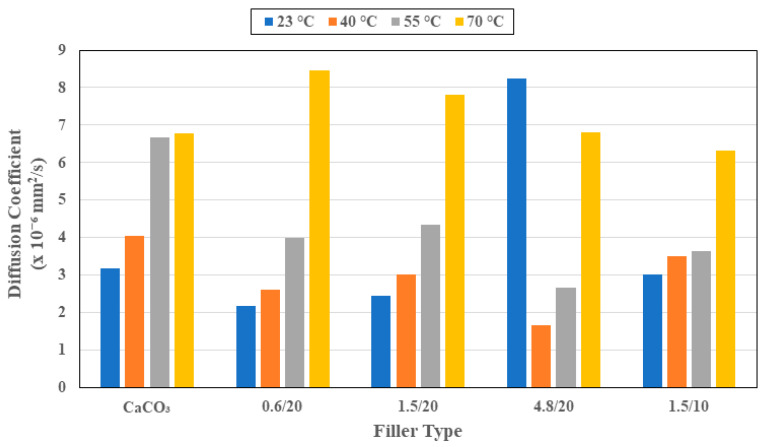
Diffusion coefficient as a function of filler type and temperature of immersion.

The diffusion coefficient, *D*, essentially represents the rate of uptake in the first stage. As can be seen from the slopes of the moisture uptake curves in Figure 1a–d and Figure 4, *D* increases with the temperature of immersion for all specimens, with the largest increase over the range being seen in the system with 0.6 μm organoclay particles, wherein the diffusion coefficient quadrupled from a value of 2.16 × 10^−6^ mm^2^/s to 8.45 × 10^−6^ mm^2^/s. In comparison, the lowest is for the system with 1.5 μm organoclay particles at 10% loading. Overall, the lowest level of *D*, with the exception of the highest temperature of immersion, is from the use of the 4.8 μm organoclay particles, indicating the greater tortuosity and blocking of moisture paths through use of the larger sized particles. It is noteworthy that the diffusion coefficients for the systems with 1.5 μm organoclay particles show different trends at the two lower temperatures where the 20% loading is more effective and the two higher temperatures where the 10% loading is more effective. This perhaps indicates the complex interaction between the greater resin–organoclay surface interaction, cure progression, and deterioration between the intercalated layers as a function of the temperature and surface area of the interaction, as the amount of water adsorbed by the clay depends on the total exposed surface area of the platelets. It is noted that the existence of multi-layer stacks with an effective exposed surface area that is less than that of the total area of the exfoliated clay particles leads to deviation from the expected linear relationship between the loading level and uptake and diffusion coefficient [50]. This can be further assessed through determination of the activation energy, *E_a_*, that must be overcome for moisture uptake as represented by an Arrhenius type relationship where

(6) $$D=D_{0}exp\left(\frac{-E_{a}}{RT}\right)$$

where *D*_0_ is a constant, *R* is the universal gas constant (=8.3143 J/mol K), and *T* is the temperature of immersion in degrees Kelvin. Plotting *ln*(*D*) versus (*1/T*) values of the activation energy of the composite with calcium carbonate fillers can be determined to be 15.1 kJ/mol K, in comparison to systems with 0.6 μm, 1.5 μm, and 4.8 μm organoclay particles at 20% loading being 23.9 kJ/mol K, 20.5 kJ/mol K, and 36.5 kJ/mol K, while that of the system with 1.5 μm particles at 10% loading is 11.9 kJ/mol K, indicating significantly higher thresholds for the systems with organoclay particles at 20% loading with the highest threshold being for the largest particle size of 4.8 μm, as would be expected from the earlier conclusions based directly on diffusion coefficients.

**Figure 5:**
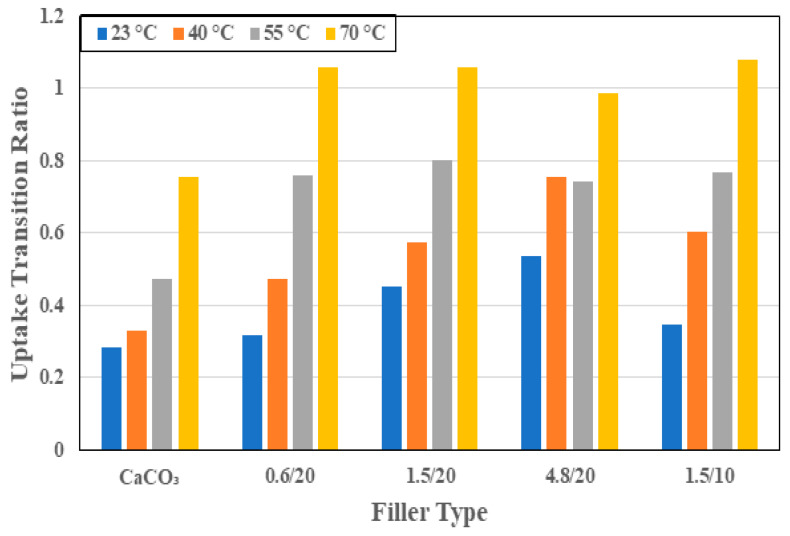
Uptake transition ratio as a function of filler type and temperature of immersion.

In the two-stage model represented by Equation (4), the first stage is diffusion dominated, whereas the second stage is representative of the slower relaxation and deterioration dominated processes. The point of transition, *M_trans_*, effectively differentiates between the two processes with an earlier attainment of transition indicating a shorter diffusion-based stage and a longer non-Fickian stage. This also indicates longer periods, in terms of levels of moisture uptake, of competition between the effects of swelling, adsorption, changes in resin- and interface-morphology, interface deterioration, and the leaching of particles and lower molecular weight species. This extent is represented by the transition ratio with lower values indicating shorter stage I regimes. As can be seen from Figure 5, the transition ratio increases for all specimens as a function of temperature, indicating increases in the extent of the diffusion dominated regime with temperature. The smallest extent is seen with the calcium carbonate fillers, indicating an earlier transition to the second stage with deterioration because of the increased interfacial debonding between particles and resin. This is an important conclusion that is related to the lower effectiveness of CaCO_3_ as compared to montmorillonite. At the two lower temperatures, the composite with 4.8 μm organoclay particles has the highest levels of transition of 53.4% and 75.5%, respectively, of the overall level of moisture uptake, whereas at the two highest temperatures of immersion of 55 °C and 70 °C, the 1.5 μm filled composites at 10% loading are the longest. Given these interactions, it is of interest to compare activation energies of transition, with the lowest activation energy of transition representing the greater propensity to stay within a diffusion dominated, i.e., stage I, regime. The activation energy of transition for the CaCO_3_ filler-based E-glass composites is 17.6 kJ/mol K, whereas that of the organoclay filled composites at the same filler loading level (20%) are 22.1 kJ/mol K, 15.6 kJ/mol K, and 9.9 kJ/mol K for the 0.6 μm, 1.5 μm, and 4.8 μm sized filler systems, respectively, whereas that for the E-glass composite with 1.5 μm organoclay particles at 10% loading is 19.9 kJ/mol K, clearly indicating that the 4.8 μm particle-based system remains diffusion dominated for the longest extent of uptake, whereas the 0.6 μm system has the least extent. It must be emphasized that the metric of focus here is not time but the level of uptake due to the diffusion dominated regime. The greater this extent, the more stable the system, in that the mechanisms are those of conventional uptake. These mechanisms are free water dominated rather than dominated through other mechanisms, which are largely irreversible and representative of deterioration and permanent damage. These other mechanisms would also affect mechanical performance to a greater extent.

**Figure 6:**
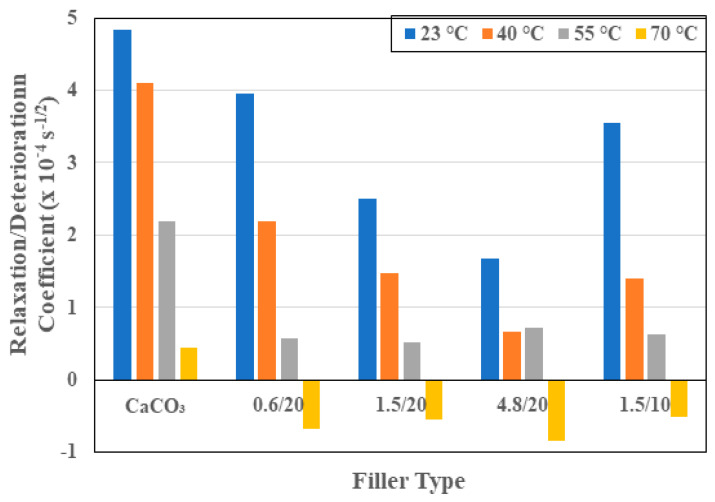
Relaxation/deterioration coefficient as a function of filler type and temperature of immersion.

The inclusion of the relaxation/deterioration parameter, *k* in Equation (4) allows for the assessment of effects of multiple mechanisms that occur over a longer time span and can involve free and bound water at different stages of uptake history, including in cracks, voids, and in interfacial regions as hypothesized by Bone et al. [51] and further elucidated by Karbhari et al. as a function of uptake related to temperature effects [52]. As seen in Figure 6, the relaxation/deterioration coefficient decreases as a function of the temperature of immersion for all material sets. The decrease is also a function of the increase in particle size and is inversely related to particle loading level. The values of *k* are, in general, lower for all the sets with organoclay particles in comparison to the set with calcium carbonate fillers, emphasizing the increased effectiveness of the organoclays through better bonding with the resin, as well as stability of these silicaceous materials over longer periods of time and levels of uptake. Activation energies of *k* are determined to be −8 J/mol K for the set with calcium carbonate fillers, −8.4 J/mol K, −5.4 J/mol K, and −4 J/mol K for the organoclay particle filled systems with 0.6 μm, 1.5 μm, and 4.8 μm particles at 20% loading, and 7.1 J/mol K for the composite with 0.6 μm organoclay fillers at 10% loading. The negative activation energies in this case imply that the rate of adsorption decreases with increases in temperature, which leads to a reduction in the probability of molecules moving between the two states of free and fixed water. Given that the transition in dominance between the diffusion and relaxation/deterioration regimes is characterized by the transition point in the uptake curve, and more effectively by the transition ratio, it is of interest to assess *k* in terms of this parameter through a linear relationship of the following form:

(7) $$k=m\left(\frac{M_{trans}}{M_{max}}\right)+k_{0}\;$$

where *m* is the slope and reflects the rate of change in the relaxation/deterioration coefficient with a decrease in the extent of the stage-II part of the uptake curve, i.e., *k* decreases as expected, as the diffusion dominated regime (stage I) occupies a greater percentage of the overall uptake. The highest rate of change, i.e., slope *m*, is seen to be from the set with calcium carbonate fillers, with the slope being 1.51–1.86 times that of the specimens with organoclay particles, where slopes were fairly close across all particle sizes. The highest slope of the organoclay set was from the 1.5 μm particles at 20% loading, with the lowest being from the 0.6 μm particles. The relative similarity among the organoclay filled systems reinforces the earlier observation of a marked difference in adsorption response between the organoclays and the conventional CaCO_3_ fillers, and emphasizes the relative advantage of replacing CaCO_3_ fillers with the organoclay particles.

### 3.2. Mechanical Characterization

The effect of changes in filler type from the conventional CaCO_3_ particles to the novel organoclays, as well as the effect of organoclay particle size and loading level on mechanical characteristics, is assessed through tensile, flexural, and short beam shear tests. The results for the unexposed specimens are listed in Table 1. Since the intent was to also focus on the hygrothermal durability of these systems using four temperatures of immersion for periods up to 1 year, the results for the specimens subject to immersion are discussed in this paper in terms of normalized values (value determined after a period of immersion at a specific temperature divided by the unexposed performance level for that set) to enable comparison across systems based on levels of retention. This allows for the efficacy of systems in terms of durability to be evaluated irrespective of the initial unexposed value.

#### 3.2.1. Tensile Characterization

**Figure 7:**
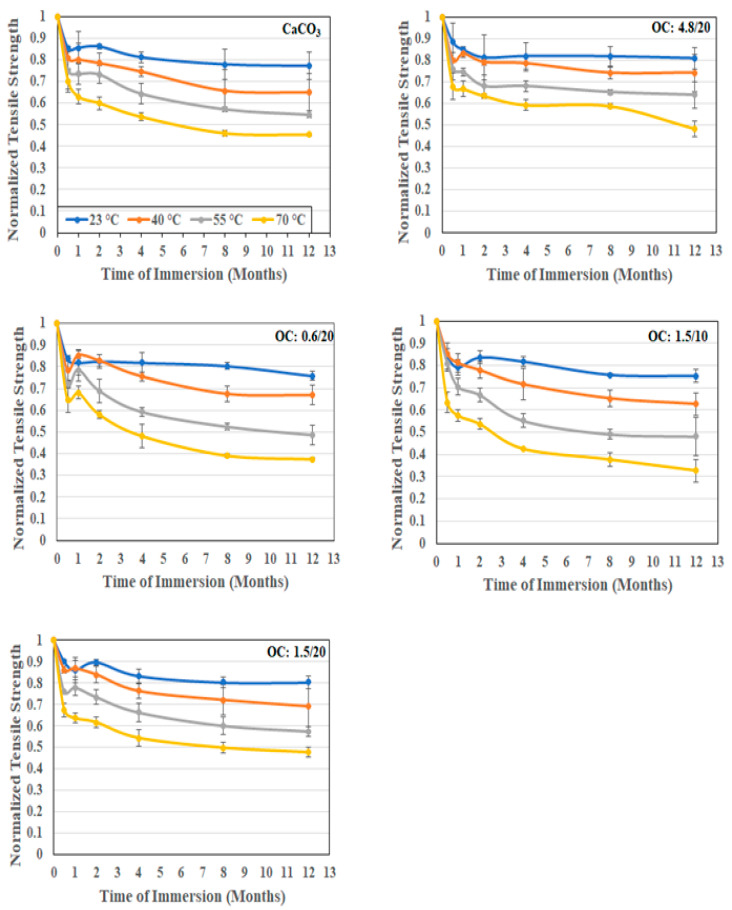
Normalized tensile strength as a function of time and temperature of immersion in deionized water.

**Figure 8:**
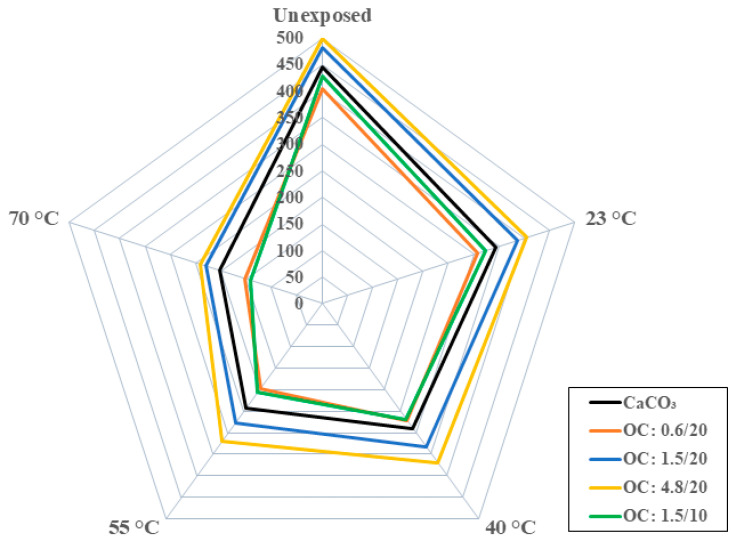
Comparison of tensile strength variation as a function of filler type at different temperatures of immersion. Scale is in MPa.

As seen in Table 1, the tensile strength of the composites with organoclay fillers increases with particle size and with loading level. In addition, the composites with 1.5 μm and 4.8 μm organoclay particles show increases above the level of the strength of the baseline CaCO_3_ filled composite. The highest increase over the CaCO_3_ filled composite system is from the 4.8 μm organoclay system at 12.2%. The increase in loading of the 1.5 μm organoclay particles from 10% to 20% loading also resulted in an increase of 12.2% with the increase over the CaCO_3_-based system being 8.02% and 12.2% at the 1.5 μm and 4.8 μm particle sizes, respectively. This increase with size and loading is in line with results reported earlier by Yilmaz et al. [53]. As shown in Figure 7, the tensile strengths for all the composite systems that were considered decrease with the time and temperature of immersion in water, with the greatest drop being at the highest temperature of immersion. The response is generically a rapid drop being in the first two weeks to a month of immersion, followed at times by a slight increase within that period due to posture processes followed by a slower decrease to an asymptotic level over longer periods of immersion. The largest drop in strength is seen in the systems with 1.5 μm organoclay filler particles at 10% loading with the level of loss at the end of 12 months being 24.7%, 37.4%, 51.9%, and 67.2% at immersion temperatures of 23 °C, 40 °C, 55 °C, and 70 °C, respectively. In comparison, the least degradation is noted in the composite with 4.8 μm organoclay fillers, wherein the drop is 19.1%, 25.8%, 36.2%, and 51.8% at the end of 12 months of immersion in water at 23 °C, 40 °C, 55 °C, and 70 °C, respectively. A graphical representation of the comparative levels of the five different composites and the deterioration at the end of 12 months of immersion at the four different temperatures is shown in Figure 8 for ease of reference. The eccentricity in the radar plot emphasizes the increased deterioration of some systems.

**Figure 9:**
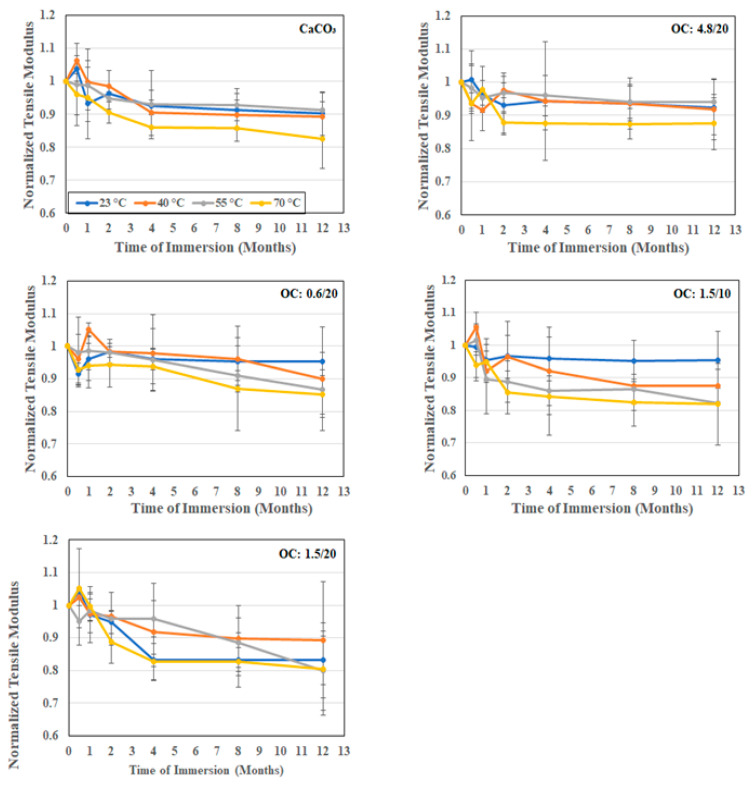
Normalized tensile modulus as a function of time and temperature of immersion in deionized water.

Given that the composites are unidirectional in terms of fiber reinforcement orientation, it is expected that fillers themselves would not substantially affect the modulus unless there was a significant loss in bond integrity between the resin and E-glass fibers, or if the particles were large enough, either individually or in agglomerated form, to cause deviations in fiber orientation, neither of which is seen in these composites. Thus, the modification in the type and size of filler would not be expected to result in substantial changes to the tensile modulus either. As seen in Table 1, the differences in moduli based on particle size and loading are almost imperceptible and largely within scatter bounds. A minor increase is seen with an increase in the size of the organoclay filler particles. As seen in Figure 9, and as would be expected, the drop in the modulus with the time and temperature of immersion is also much lower than that noted for tensile strength, with the greatest drop being after immersion at 70 °C, wherein the calcium carbonate filled system showed a 17.5% drop, and the composites with organoclay fillers at the same loading level, 20%, showing drops of 14.9%, 19.5%, and 13.3% for the composites with 0.6 μm, 1.5 μm, and 4.8 μm organoclay particles, respectively. A decrease in the loading level of the 1.5 μm particles from 20% to 10% resulted in a very small decrease in the drop as well, from 19.5% to 18.1%, which is well within the scatter bounds but suggests that the drop in the modulus is likely due to loss in the interfacial bond between the particles and the bulk resin, in addition to the expected loss between the fiber and matrix. The layered structure of the organoclay particles causes a significant enhancement in barrier type characteristics due to the interfacial interactions at the layer level with intercalation of the polymer into the organoclay galleries, which would result in better overall integrity [19,43,54,55,56,57].

#### 3.2.2. Flexural Response

**Figure 10:**
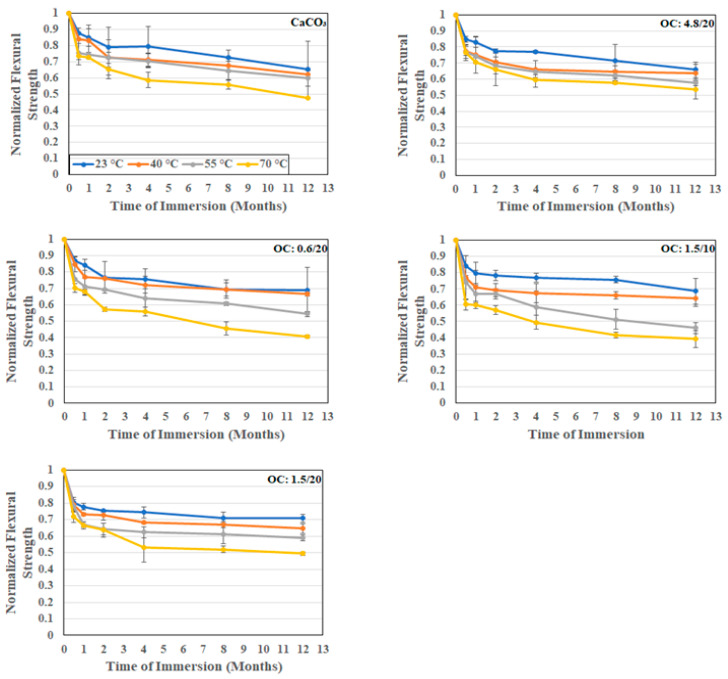
Normalized flexural strength as a function of time and temperature of immersion in deionized water.

As seen in Table 1, the flexural strength increases by a small amount both with the increase in the particle size of the organoclay fillers and the loading level, with the best performance being using the 4.8 μm particles at 20% loading, which represents a 6% improvement over the baseline E-glass composites with the calcium carbonate filler. As can be seen in Figure 10, there is an initial rapid drop in flexural strength because of moisture uptake, followed by a slower progression with the time of immersion. The increase in temperature between 23 °C and 40 °C is not seen to have a significant change, whereas the change due to the further increase in the temperature of immersion is far greater. Specimens tested at the two lower temperatures did not show significant interfacial cracking, whereas those subject to higher temperatures showed interlaminar splits. The maximum degradation, as expected, is at the end of the 12-month period at 70 °C with deterioration levels of 59.4%, 50.6%, and 46.3% for the composites with organoclay fillers at 20% loading for particle sizes of 0.6 μm, 1.5 μm, and 4.8 μm, respectively. In comparison, the composite with CaCO_3_ particle fillers indicated a 52.7% drop. A decrease in the loading level for the composites with 1.5 μm organoclay fillers from 20% to 10% increased the deterioration substantially from 50.6% to 60.8%. It is noted that previous research on organoclay fillers indicated that an increase in the surface area of the filler not only enhances bonding with the bulk resin but also enhances the stress transfer to fibers in compression [43,57,58], which provides an explanation for the current response trends, with less splitting towards the compression face in the composites with organoclay fillers. It is noted that the tests were conducted in the four-point flexure mode and failures across all specimens were seen to be similar, with an increase in the interlayer separation with increasing time and temperature of immersion.

#### 3.2.3. Interfacial Strength

**Figure 11:**
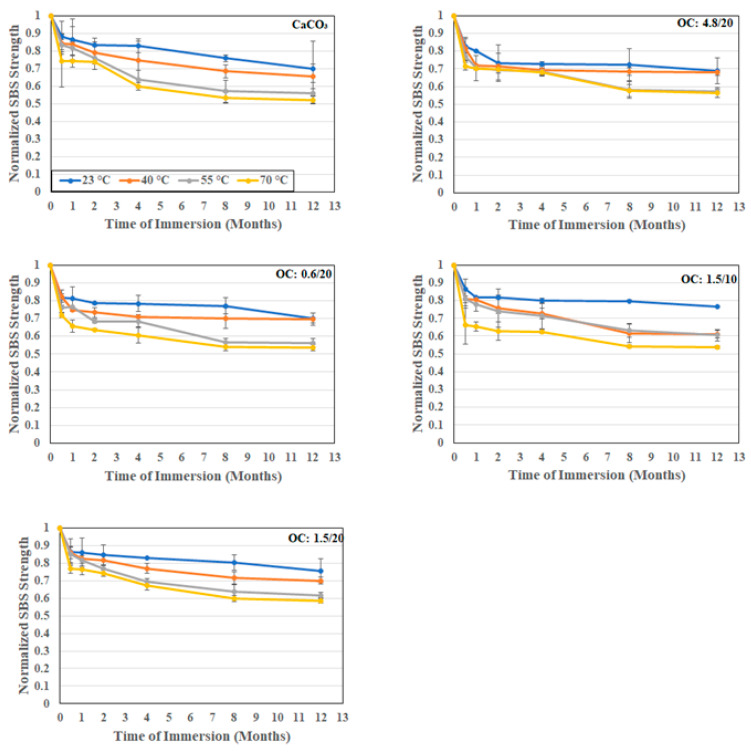
Normalized longitudinal SBS strength as a function of time and temperature of immersion in deionized water.

**Figure 12:**
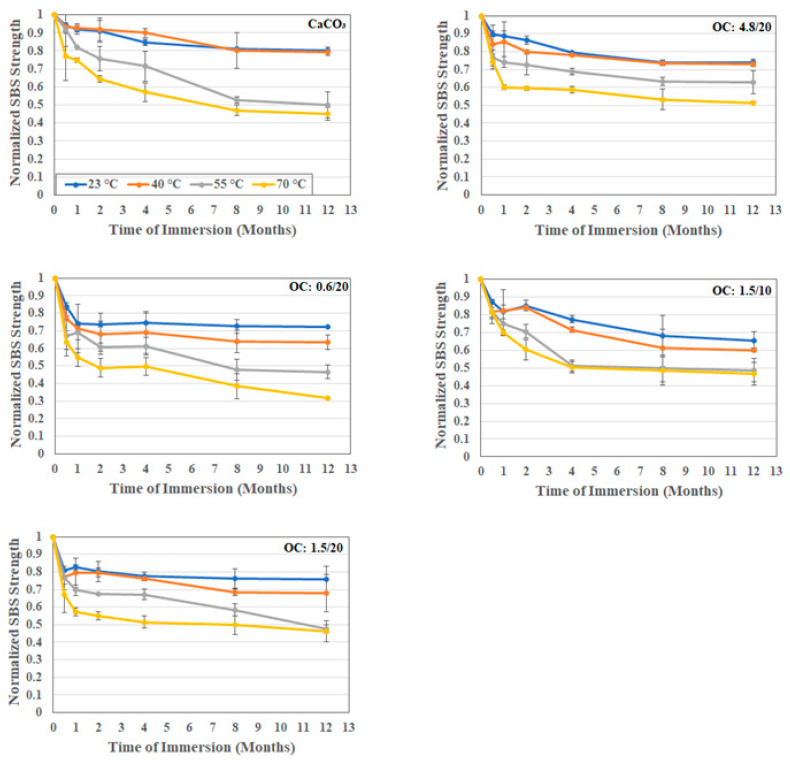
Normalized transverse SBS strength as a function of time and temperature of immersion in deionized water.

As was noted earlier, the morphology and structure of the filler particles play a role in interfacial bonding and uptake kinetics. The short beam shear test has been previously shown to be a powerful tool for the assessment of the effects of the moisture-associated durability of composites [59,60]. In the current investigation, since the focus is on particles used as fillers rather than the E-glass fibers themselves, tests were conducted in both the longitudinal and transverse directions so that the comparison between these would more clearly identify the effect of the particles, since these would be additive in the former and the primary differentiator in the latter. As noted in Table 1 and Figure 11 and Figure 12, for the longitudinal and transverse directions, respectively, the incorporation of organoclay particles results in a greater increase over that through use of calcium carbonate fillers.

The level of increase is greater with loading level but mixed as related to particle size. In the longitudinal case, the highest increase, 9.7%, is seen with the 1.5 μm particles, with decreases observed as the size of the particles increase. It is interesting to note that the levels attained using the 0.6 μm and 4.8 μm particles are within the bounds of scatter. However, in the case of transverse directionality, the level of increase decreases with the increase in the diameter of filler particles, which is similar to results reported by Boukhili et al. [11] and in line with the expected effect of the greater overall surface area for bonding and increased moisture path tortuosity with the use of the smaller particles.

**Figure 13:**
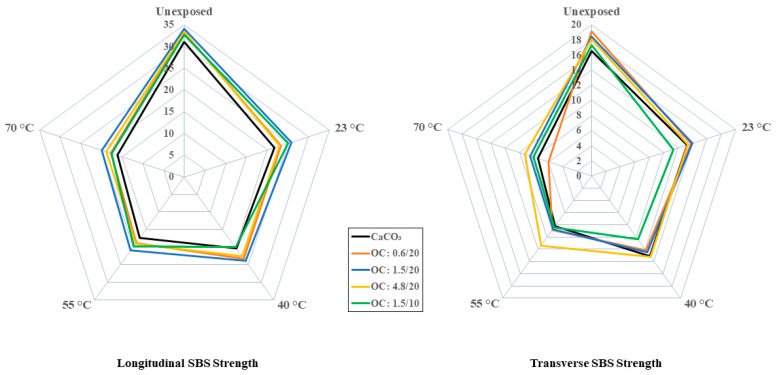
Comparison of SBS strength variation as a function of filler type at different temperatures of immersion. Scale is in MPa.

In all cases, the effect of the use of organoclay particles provides a positive enhancement over that of calcium carbonate, with the level of increase over the composite with calcium carbonate fillers being 15.7%, 11.6%, and 9.6% for the 0.6 μm, 1.5 μm, and 4.8 μm particles at 20% loading. Interestingly the lowest level of increase in the transverse direction is similar to the greatest level of increase, 9.7%, in the longitudinal direction. As would be expected, the drop as a function of time and temperature is greater in the transverse direction due to the decreased influence of fibers in that direction. Unlike in other cases where the extent of drop in performance increases with temperature, in this case, the difference between the two highest temperature levels is far less, emphasizing the presence of a temperature threshold at which the change in mechanisms occurs. A comparison of the overall effects of temperature in the longitudinal and transverse directions can be seen in Figure 13 which emphasizes the greater clarity in assessing differences in interfacial response due to hydrothermal exposure through tests in the transverse direction. It is noted that the moisture uptake curves all demonstrated a consistent increasing trend over the full period of immersion at 23 °C and 40 °C, with minor decreases being noted towards the end of the period of immersion at 50 °C and substantially earlier at 70 °C. The level of deterioration in transverse short beam shear strength is the least for the 4.8 μm system at 51.6% and the greatest for the 0.6 μm system at 68.5%, which interestingly coincides with the ranking of activation energies for the relaxation/deterioration coefficient, *k*, since the changes in interfacial strength characteristics are representative of longer term, irreversible effects that would occur over the longer periods of immersion [60] and hence would be noted during stage II of the moisture uptake curves.

## 4. Summary and Conclusions

This investigation focused on a pultruded E-glass/vinylester composite with montmorillonite organoclay fillers added at loading levels of 10% and 20%, with a focus on assessing the effects on moisture uptake and mechanical performance characteristics. It is seen that the uptake is non-Fickian in all cases, with the level of uptake increasing with the time of immersion and without reaching saturation over the 1-year period of investigation. A two-stage model that incorporates an initial diffusion dominated regime and a second slower relaxation/deterioration regime is shown to model this behavior well in relation to the increasing trends and to decreasing uptake due to the leaching of lower molecular weight species and fillers. The addition of the organoclays is shown to positively enhance composite response by substantially decreasing uptake levels and enhancing overall stability. Diffusion coefficients are seen to increase with temperature, with the coefficients for the composites with organoclay fillers being substantially lower than those for the conventional calcium carbonate system, emphasizing the positive enhancement of the replacement primarily due to the layered structure of organoclay particles, which cause enhancement in barrier type characteristics due to the better overall interfacial integrity and greater resulting tortuosity of the path of sorbed water. A comparison of the ranking of mechanical characteristics of the systems in terms of the percentage of retention of unexposed characteristics after immersion for 1 year in deionized water at the highest temperature used in the investigation is given in Table 2.

Overall, the composite with the 4.8 μm organoclay fillers is seen to have the best performance at the highest temperatures of immersion (which, in terms of a time–temperature superposition methodology, would suggest the best long-term durability and service-life). The particle size is hypothesized to provide a greater interaction at the layer level, with the intercalation of the polymer into the organoclay galleries resulting in better resistance to moisture-induced deterioration and hence resulting in enhanced hygrothermal durability. It is also the only system where the performance retention remained above 50% irrespective of the time and temperature of immersion and type of mechanical characteristic investigated. The increased level of performance of the organoclay filler system over the conventional calcium carbonate system at the unexposed level, in conjunction with the better response over the time and temperature of immersion, indicate that these novel materials can be used at desired loading levels that would provide multifunctional properties of interest and thus raise the potential for the further development of these novel systems that incorporate fillers that are not only extenders and process aids but also serve to enhance mechanical characteristics and durability under harsh conditions at low cost. This research serves as the foundational step in further investigations into the role of organoclay particles in enhancing multifunctionality and durability. Ongoing and future research will focus on developing an increased understanding of the relation between moisture uptake and changes in mechanical characteristics. A detailed study of dynamic thermal mechanical analysis (DMTA) characterization is also expected to provide insight into the effects of moisture and organoclay particle size on glass transition temperature and dynamic moduli with the aim of better predicting long-term response and to establish design thresholds.

## Figures and Tables

**Table 1:** Unexposed performance characteristics (CacO_3_: calcium carbonate; OC: organoclay).

Characteristic	Units	Filler Type				
		CaCO_3_	OC: 0.6/20	OC: 1.5/20	OC: 4.8/20	OC: 1.5/10
**Tensile Strength**	MPa	445.26 [9.93]	412.03 [19.51]	480.98 [23.30]	499.66 [14.92]	428.51 [17.79]
**Tensile Modulus**	GPa	30.97 [2.00]	30.88 [1.01]	31.15 [1.89]	31.62 [1.85]	30.69 [1.31]
**Flexural Strength**	MPa	460.71 [50.14]	470.29 [40.69]	486.81 [22.78]	488.42 [38.51]	482.28 [29.04]
**Longitudinal SBS Strength**	MPa	31.04 [4.94]	33.28 [1.83]	34.04 [2.75]	33.33 [1.72]	32.73 [3.37]
**Transverse SBS Strength**	MPa	16.53 [1.90]	19.12 [0.30]	18.44 [1.10]	18.11 [0.98]	17.26 [1.17]

**Table 2:** Ranking of systems based on percentage retention (1 has the highest retention and 5 has the lowest). Numbers in red indicate that the 50% threshold retention has been exceeded.

Temperature of Immersion (°C)	Performance Characteristic	Filler Type				
		*CaCO* *_3_*	*OC: 0.6/20*	*OC: 1.5/20*	*OC: 4.8/20*	*OC: 1.5/10*
70	Tensile Strength	3	4	2	1	5
	Tensile Modulus	3	2	5	1	4
	Flexural Strength	**3**	**4**	**2**	1	**5**
	SBS Strength (L)	5	4	1	2	3
	SBS Strength (T)	**4**	**5**	**3**	1	**2**

## Data Availability

The original contributions presented in the study are included in the article, further inquiries can be directed to the corresponding author.
